# Round Shoulders in Girls

**Published:** 1897-11

**Authors:** 


### ROUND SHOULDERS IN GIRLS.

Dr. E. H. Bradford (*Boston Medical and Surgical Journal*, Sept. 9, 1897) discusses a common cause of round shoulders in girls. He says, both for convenience and owing to an exaggerated fear of injury to the pelvis, it is the custom, in dressing children, to throw the weight of the garments upon the shoulders, fastening the skirts, and in many instances stockings, to the waist These waists are made of various shapes, but usually have a narrow shoulder strap, which presses upon the upper portion of the trapezius, and upon the clavicles to the inner side of the head of the humerus. They also exert pressure, if pulled downward, upon the upper portion of the sternum. Where the skirts are buttoned to the front of this waist as is usually the case, a considerable drag is exerted, and in this way pressure falls upon the trapezius and the sternum, the head is thrown forward, the shoulders drop forward, and the chest is contracted. The amount of weight of the clothing is not inconsiderable, making the erect position less easily borne, the child naturally seeking the position of greatest comfort. Where stocking-supporters are added the downward drag may become more than would be supposed. Where a faulty position is assumed a greater part of the day, the soft tissues adapt themselves, and a faulty attitude becomes habitual, the ligaments adapting themselves to such a shape. It is not probable that pressure upon clavicles is exerted which would cause a marked distortion, as these are particularly strong bones, though this may occur in rachitic children. Fortunately, however, in rachitic children, when rickets is most prevalent, the weight of the garments is not great. Somewhat the same effect may result from the use of suspenders or from the straps of an anteroposterior support in Pott's disease. Suspenders, fortunately, are not worn ordinarily until the figure assumes its shape.

The prevention of this deformity is more important than the cure, which must necessarily involve a long-continued treatment, designed to stretch the ligaments and strengthen the muscles. It is important to bear in mind that the clothing should be so arranged that none of the weight of the skirt or underwear should drag upon the front of the waist. As far as possible, where any weight is thrown upon the shoulders, it should be a force that would pull the shoulders back. The waist should be so constructed as to make no pressure upon the sternum. The shoulder-straps should not bear upon the middle of the clavicles, but upon the tip of the acromion. In young, growing children most of the weight of the underwear should be borne upon the hips, lessening the load upon the shoulders.

